# Un cas de syndrome de Guillain-Barré dû à une infection par *Rickettsia conorii* en Tunisie

**DOI:** 10.48327/mtsi.v5i4.2025.768

**Published:** 2025-10-22

**Authors:** Yosr BEN TAHER, Rania AMMAR, Mabrouk BAHLOUL, Chokri BEN HAMIDA

**Affiliations:** 1 Service de réanimation médicale du Centre hospitalo-universitaire (CHU) Habib Bourguiba de Sfax, Tunisie; 2 Université de Sfax, Tunisie

**Keywords:** Fièvre boutonneuse méditerranéenne,, Rickettsia conorii,, Polyradiculonévrite aiguë, Syndrome de Guillain-Barré, Tique, Tunisie, Afrique du Nord, Mediterranean spotted fever, *Rickettsia conorii*, Acute polyradiculoneuritis, Guillain-Barre syndrome, Tick, Tunisia, North Africa

## Abstract

**Introduction:**

La fièvre boutonneuse méditerranéenne, causée par *Rickettsia conorii,* est une infection endémique dans le bassin méditerranéen, transmise principalement par les tiques. L’association entre cette infection et le syndrome de Guillain-Barré (SGB) est rare.

**Observation:**

Nous rapportons le cas d’un patient tunisien de 29 ans, sans antécédents médicaux, présentant une quadriplégie flasque évoluant vers une détresse respiratoire, associée à une fièvre persistante et une hépatosplénomégalie. Le diagnostic de SGB sous sa forme axonale motrice aiguë, a été confirmé par électroneuromyogramme et l’infection à *R. conorii* par la sérologie. Le traitement par immunoglobulines et doxycycline a conduit à une amélioration clinique progressive.

**Conclusion:**

Ce cas souligne l’importance de penser à l’infection à *R. conorii,* devant un SGB associé à des signes systémiques en particulier dans un pays endémique comme la Tunisie.

## Abstract (en)

**Introduction:**

Mediterranean spotted fever is an infection caused by *Rickettsia conorii* that is endemic to the Mediterranean basin. It is mainly transmitted by ticks. The association between this infection and Guillain-Barre syndrome (GBS) is uncommon.

**Observation:**

We present the case of a 29-year-old Tunisian patient with no prior medical conditions who presented with flaccid quadriplegia that progressed to respiratory distress. This was accompanied by persistent fever and hepatosplenomegaly. Acute motor axonal GBS was diagnosed through electroneuromyography, and *R. conorii* infection was diagnosed through serology. Treatment with immunoglobulins and doxycycline led to gradual clinical improvement.

**Conclusion:**

This case underscores the importance of considering *R. conorii* infection in patients with GBS presenting with systemic symptoms, especially in endemic regions like Tunisia.

## Introduction

L’infection à *Rickettsia conorii* est endémique dans le bassin méditerranéen, où elle est appelée la fièvre boutonneuse méditerranéenne (FBM) [11. 16] . C’est une infection transmise principalement par la tique du chien *Rhipicepalus sanguineus* [11,16]. Dans la littérature, l’association du syndrome de Guillain-Barré (SGB) ou de la polyradiculonévrite aiguë avec l’infection à *R. conorii* est exceptionnelle [15]. Quelques cas ont néanmoins été décrits dans la littérature [4,5,10,12,16] . Nous rapportons un cas d’une infection à *R. conorii* associée au SGB en Tunisie.

## Observation

Monsieur MA, âgé de 29 ans, originaire du sud de la Tunisie, sans antécédents médicaux notables, est agriculteur et éleveur de moutons. Il ne possède pas de chien lui-même mais ses voisins en détiennent un. Il a été hospitalisé initialement dans le service de neurologie pour une paraplégie flasque. Son histoire de la maladie remonte à 11 jours avec apparition d’une fièvre chiffrée à 40 °C traitée initialement par des antipyrétiques prescrits par son médecin généraliste. L’évolution a été marquée par l’installation d’une quadriplégie flasque 10 jours après le début de la fièvre, avec installation concomitante de troubles de la déglutition. Deux jours après, une détresse respiratoire a nécessité une prise en charge en réanimation et une ventilation mécanique. L’examen en réanimation a montré un patient fébrile à 39 °C, une pression artérielle à 120/80 mm Hg, un pouls dissocié avec 84 battements par minute. L’examen abdominal objectivait une hépatosplénomégalie, avec une flèche hépatique mesurée à 19 cm et une flèche splénique à 18 cm. L’examen neurologique montrait une tétraplégie flasque avec abolition des réflexes ostéotendineux. Il n’y avait pas de lésion d’escarre ni d’éruption cutanée. L’analyse du liquide céphalorachidien a montré une cytologie à 5 éléments de la lignée blanche, une glycorachie à 3,8 mmol/l (rapport glycorachie/glycémie sanguine = 0,67), une protéinorachie à 0,26 g/l. L’éléctroneuromyogramme révélait une forme axonale motrice pure du SGB. L’IRM médullaire était sans anomalie. Le patient a reçu une cure d’immunoglobulines G (IgG) à raison d’une dose de 0,4 g/kg/j pendant 5 jours soit 30 g/j.

Le bilan biologique montrait une anémie inflammatoire (hémoglobine à 6,1 g/dl, hypochrome microcytaire arégénérative, volume globulaire moyen à 67 fl, réticulocytes à 50 400/mm^3^, un fer sérique bas à 5 µmol/l et une ferritinémie normale à 280 ng/ml), des globules blancs à 4 300 leucocytes/mm^3^ et une protéine C-réactive élevée à 77 mg/l. La radiographie thoracique révélait un syndrome interstitiel basal. Les sérologies des germes atypiques (*Coxiella*, *Mycoplasma*, *Legionnella)* étaient négatives, ainsi que les sérologies de Wright, *Bartonella,* hépatites virales B et C et VIH et la sérologie du West Nile. La sérologie pour *R. conorii* par immunofluorescence indirecte montrait une réponse IgM positive (> 1/512) (valeur normale < 1/128) et IgG < 1/128 (négative). Le patient a été traité par doxycycline à la dose de 200 mg/j pendant 14 jours, avec une apyrexie obtenue dès le début du traitement. Un contrôle sérologique après 15 jours révélait une élévation significative des IgM pour *R. conorii* à 1/2 048 avec des IgG toujours négatives. L’évolution clinique était favorable avec un sevrage de la ventilation mécanique sur canule de trachéotomie et une récupération progressive de sa motricité surtout au niveau des membres supérieurs. Il était autorisé à sortir après 34 jours d>hospitalisation en réanimation après décanulation, avec des séances de kinésithérapie. Aucun contrôle sérologique n’a été fait après la sortie de l’hôpital.

## Discussion

Nous décrivons le premier cas de SGB en lien avec une FBM à *R. conorii* en Tunisie. L’infection à *Rickettsia* est souvent transmise par des tiques du chien de l’espèce *Rh. sanguineus* [1,11], en particulier dans les zones endémiques comme le bassin méditerranéen, l’Afrique du Nord et le Maghreb [11,15]. La Tunisie est une zone endémique. La première description de *Rickettsia* en Tunisie a été faite par Conor et Brush en 1925 [8]. À notre connaissance, aucune étude tunisienne récente n’a porté sur la FBM. Dans une série algérienne (de 2012 à 2014), dans la wilaya de Tizi-Ouzou, 310 patients ayant présenté une fièvre éruptive ont été colligés. Le diagnostic de rickettsiose par la sérologie par immunofluorescence indirecte (IFI) a été confirmé chez 144 patients (46,5%). Il s’agissait de *R. conorii* dans 127 cas (65,1%), de *R. felis* dans 18 cas (9,2%) et de *R. typhi* dans 5 cas (2,6%) [2].

La FBM se manifeste par une fièvre prolongée comme chez notre patient, une éruption cutanée maculo-papuleuse pouvant toucher la paume des mains et la plante des pieds, une escarre d’inoculation et des signes systémiques tels qu’une hépatosplénomégalie et plus rarement une pneumopathie [13]. Cependant, chez notre patient, il n’a pas été trouvé de lésion cutanée. L’infection à *Rickettsia* peut affecter plusieurs organes, incluant le système nerveux central [14].

Les manifestations neurologiques les plus courantes signalées dans les infections par les rickettsies sont la méningite, l’encéphalite et l’encéphalomyélite aiguë disséminée [6]. Une paralysie faciale unilatérale [10], une quadriplégie flasque [4], un accident vasculaire [3] ou une perte de la vision [9] peuvent aussi être observés [15]. Le premier cas de SGB secondaire à une infection à *R. conorii* a été décrit en 1999 [5]. Quelques cas dans la littérature ont décrit l’association d’un SGB [5] ou polyradiculonévrite [4,12] avec l’infection par *R. conorii* [7].

Les infections bactériennes, telles que celles causées par *Rickettsia*, peuvent déclencher une réponse inflammatoire systémique susceptible de perturber le système nerveux périphérique [12]. Un mécanisme dysimmunitaire pourrait s’ajouter au mécanisme vasculaire dans le développement de l’atteinte neurologique périphérique [12].

La théorie de mimétisme moléculaire suggère que l’auto-immunité pourrait être stimulée par des antigènes présents à la surface des *Rickettsia,* qui ressembleraient à des structures neuronales, ce qui provoquerait une réponse immunitaire amplifiée, aboutissant à une attaque de la myéline ou des axones des nerfs périphériques [12]. En Tunisie, la méthode diagnostique pratiquée est la sérologie par IFI sur des échantillons de sérum. Elle constitue la technique de référence pour le diagnostic des rickettsioses. La sérologie par immunofluorescence indirecte et surtout la séroconversion est utile pour faire le diagnostic [1,6]. Une élévation des IgM contre *R. conorii* à des taux supérieurs à 1/128 confirme le diagnostic selon les valeurs de notre laboratoire [1]. La cure d>immunoglobulines a utilisé des IgG et non des IgM, ce qui ne peut pas expliquer l’augmentation des IgM observée. Le traitement de référence de la FBM est la doxycycline chez l’adulte (200 mg par jour) [12]. Ce traitement a permis une disparition spectaculaire de la fièvre chez notre patient dès son introduction.

## Conclusion

Ce cas clinique met en lumière une association rare mais possible entre une infection à *R. conorii* et le SGB. L’identification précoce de la rickettsiose et la prise en charge appropriée peuvent améliorer le pronostic des formes graves de SGB déclenchées par cette infection.

## Consentement éclairé

Nous avons obtenu le consentement oral du patient.

## Financement

Aucun financement n’a été nécessaire.

## Contributions des auteurs et autrices

Yosr Ben Taher: conception du rapport de cas, prise en charge diagnostique et thérapeutique du patient, rédaction, révision et validation du manuscrit. Rania Ammar: prise en charge diagnostique et thérapeutique du patient, rédaction, révision et validation du manuscrit. Mabrouk Bahloul: rédaction, révision et validation du manuscrit. Chokri ben Hamida: rédaction, révision et validation du manuscrit

## Déclaration de liens d’intérêt

Aucun lien d’intérêt n’a été déclaré.
