# Supplementary material for: A case of Guillain-Barré syndrome due to infection with Rickettsia conorii in Tunisia
Source: Med Trop Sante Int. 2025 Oct 22;5(4):mtsi.v5i4.2025.768. [Article in French] doi: 10.48327/mtsi.v5i4.2025.768 (PMC12948365; doi:10.48327/mtsi.v5i4.2025.768)
Supplement: Related texts

# Research article: A case of Guillain-Barré syndrome due to infection with Rickettsia conorii in Tunisia

## Introduction

Infection with *Rickettsia conorii* is endemic to the Mediterranean basin, where it is known as Mediterranean spotted fever (MSF). This infection is primarily transmitted by the dog tick, *Rhipicephalus sanguineus* [11,16]. The association of Guillain-Barré syndrome (GBS) or acute polyradiculoneuritis with *R. conorii* infection is rare in the literature [15]. However, a few cases have been reported [4,5,10,12,16]. Here, we present a case of *R. conorii* infection associated with GBS in Tunisia.

## Observation

Mr. MA is a 29-year-old farmer and sheep breeder from southern Tunisia with no significant medical history. He does not own a dog, but his neighbors do. He was initially hospitalized in the neurology department with flaccid paraplegia. Eleven days prior, he developed a fever of 40°C, which was initially treated with antipyretics prescribed by his general practitioner. Ten days after the onset of fever, he developed flaccid quadriplegia and swallowing disorders. Two days later, he required intensive care and mechanical ventilation due to respiratory distress. Upon examination in the ICU, the patient had a fever of 39°C, a blood pressure of 120/80 mmHg, and a dissociated pulse of 84 beats per minute. An abdominal examination revealed hepatosplenomegaly, with a liver span of 19 cm and a spleen span of 18 cm. A neurological examination revealed flaccid quadriplegia and loss of deep tendon reflexes. There were no pressure sores or skin rashes. Analysis of the cerebrospinal fluid revealed a white blood cell count of 5, a glucorachia of 3.8 mmol/L (with a glucorachia-to-blood glucose ratio of 0.67), and a proteinorachia of 0.26 g/L. The electroneuromyogram revealed a pure motor axonal form of GBS. The spinal cord MRI showed no abnormalities. The patient received a five-day course of intravenous immunoglobulin (IVIG) at a dose of 0.4 g/kg/day, or 30 g/day.

Laboratory tests revealed inflammatory anemia (hemoglobin of 6.1 g/dL, hypochromic microcytic regenerative anemia, a mean corpuscular volume of 67 fL, 50,400 reticulocytes/mm^3^, low serum iron of 5 μmol/L, and normal ferritin of 280 ng/mL), 4,300 white blood cells/mm3, and elevated C-reactive protein of 77 mg/L. A chest X-ray revealed basal interstitial syndrome. Serology tests for atypical organisms *(Coxiella,* *Mycoplasma*, and *Legionella)* were negative. So were Wright serology and tests for *Bartonella*, viral hepatitis B and C, HIV and the West Nile virus. Indirect immunofluorescence serology for *R. conorii* showed a positive IgM response (>1/512) (normal value <1/128) and negative IgG (<1/128). The patient was treated with doxycycline at a dose of 200 mg per day for 14 days and became afebrile from the start of treatment. A serological check after 15 days revealed a significant increase in *R. conorii* IgM to 1/2,048, while IgG remained negative. The patient had a favorable clinical course, weaning from mechanical ventilation via tracheostomy tube, and gradual recovery of motor function, especially in the upper limbs. The patient was discharged from intensive care after 34 days, having undergone extubation and physical therapy sessions. No serological tests were performed after he was discharged.

## Discussion

We present the first reported case of GBS associated with MSF caused by *R. conorii* in Tunisia. *Rickettsia* infection is often transmitted by dog ticks of the *Rh. sanguineus* species [1,11], particularly in endemic areas such as the Mediterranean basin, North Africa, and the Maghreb [11,15]. Tunisia is an endemic area. *R. conorii* was first described in Tunisia by Conor and Brush in 1925 [8]. To our knowledge, no recent Tunisian studies have focused on MSF. In an Algerian study conducted from 2012 to 2014 in the Tizi Ouzou region, 310 patients with eruptive fever were examined. Rickettsiosis was diagnosed by indirect immunofluorescence (IIF) serology in 144 patients (46.5%). Of those, 127 cases were *R. conorii* (65.1%), 18 were *R. felis* (9.2%), and five were *R. typhi* (2.6%) [2].

MSF typically presents with a prolonged fever and a maculopapular rash that affects the palms and soles. Other symptoms include an inoculation eschar and systemic signs such as hepatosplenomegaly and, less commonly, pneumonia. However, our patient did not have any skin lesions. Rickettsial infections can affect several organs, including the central nervous system [14].

The most common neurological manifestations reported in rickettsial infections are meningitis, encephalitis, and acute disseminated encephalomyelitis [6]. Other manifestations [15] may include unilateral facial paralysis [10], flaccid quadriplegia [4], stroke [3] or vision loss [9].

The first case of GBS secondary to *R. conorii* infection was described in 1999. Several cases in the literature describe the association of GBS or polyradiculoneuritis [4,12] with *R. conorii* infection [7].

Bacterial infections, such as those caused by *Rickettsia,* can trigger a systemic inflammatory response that disrupts the peripheral nervous system [12]. Immune dysregulation could contribute to vascular mechanisms in the development of peripheral neurological damage [12].

The molecular mimicry theory suggests that autoimmunity could be stimulated by antigens on the surface of *Rickettsia* that resemble neuronal structures. This would cause an amplified immune response that attacks the myelin or axons of the peripheral nerves [12]. In Tunisia, the diagnostic method is IIF serology on serum samples. This is the gold standard technique for diagnosing rickettsiosis. Indirect immunofluorescence serology, especially seroconversion, is useful for diagnosis [1,6]. According to our laboratory values [1], an elevation of IgM against *R. conorii* to levels above 1/128 confirms the diagnosis. However, the immunoglobulin treatment used was IgG, not IgM, which cannot explain the observed increase in IgM. The standard treatment for MSF is doxycycline (200 mg per day) for adults [12]. This treatment led to a dramatic disappearance of fever in our patient as soon as it was introduced.

## Conclusion

This clinical case highlights the rare but possible link between *R. conorii* infection and GBS. Early identification of rickettsiosis and proper management can improve the prognosis of severe cases of GBS caused by this infection.

## Informed Consent

We obtained the patient’s verbal consent.

## Funding

No funding was required.

## Authors’ contributions

Yosr Ben Taher: Designed the case report; managed the patient’s diagnosis and treatment; wrote, revised, and validated the manuscript. Rania Ammar: Diagnostic and therapeutic management of the patient; writing, revision, and validation of the manuscript. Mabrouk Bahloul: Writing, revising, and validating the manuscript. Chokri Ben Hamida: Writing, revision, and validation of the manuscript.

## Conflict of interest

The authors declare no conflicts of interest related to this work.
